# Supplementary material for: Fronto-motor circuits linked to effort-based decision-making and apathy in healthy subjects
Source: Commun Biol. 2025 Aug 30;8:1320. doi: 10.1038/s42003-025-08780-8 (PMC12398607; doi:10.1038/s42003-025-08780-8)
Supplement: Supplementary file 1 — Supplementary Information [file 42003_2025_8780_MOESM1_ESM.pdf]

Supplementary Information

Fronto-motor circuits linked to effort-based decision-making and apathy in healthy subjects

Gerard Derosiere, Pierre Vassiliadis, Laurence Dricot, Quentin Dessain, Nicolas Delinte, Alexandre Zénon, Julie Duque

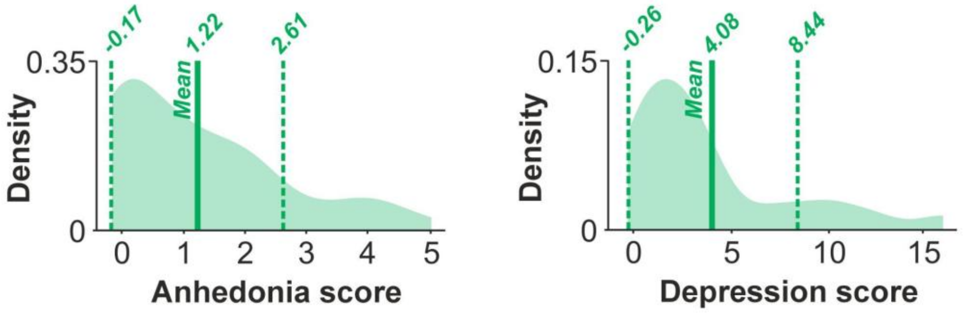

Supplementary Figure 1: Distribution of anhedonia and depression scores. We conducted a comprehensive neuropsychological assessment to quantify apathy scores, anhedonia scores and depression scores in our cohort of 45 healthy subjects. Apathy scores were assessed using the extended version of the Lille Apathy Rating Scale (LARS-e)[1], while anhedonia and depression were measured using the Snaith-Hamilton Pleasure Scale (SHAPS) and the Depression Anxiety Stress Scales (DASS), respectively. While density distributions of apathy scores are presented in the main manuscript, this figure presents these distributions for anhedonia and depression scores within the cohort. Anhedonia scores ranged from 0 to 5, with a mean of 1.22. Depression scores ranged from 0 to 16, with a mean of 4.08. These data reveal a broad range of anhedonia and depression levels across subjects, underscoring the importance of controlling for these variables through partial correlation analyses, as they

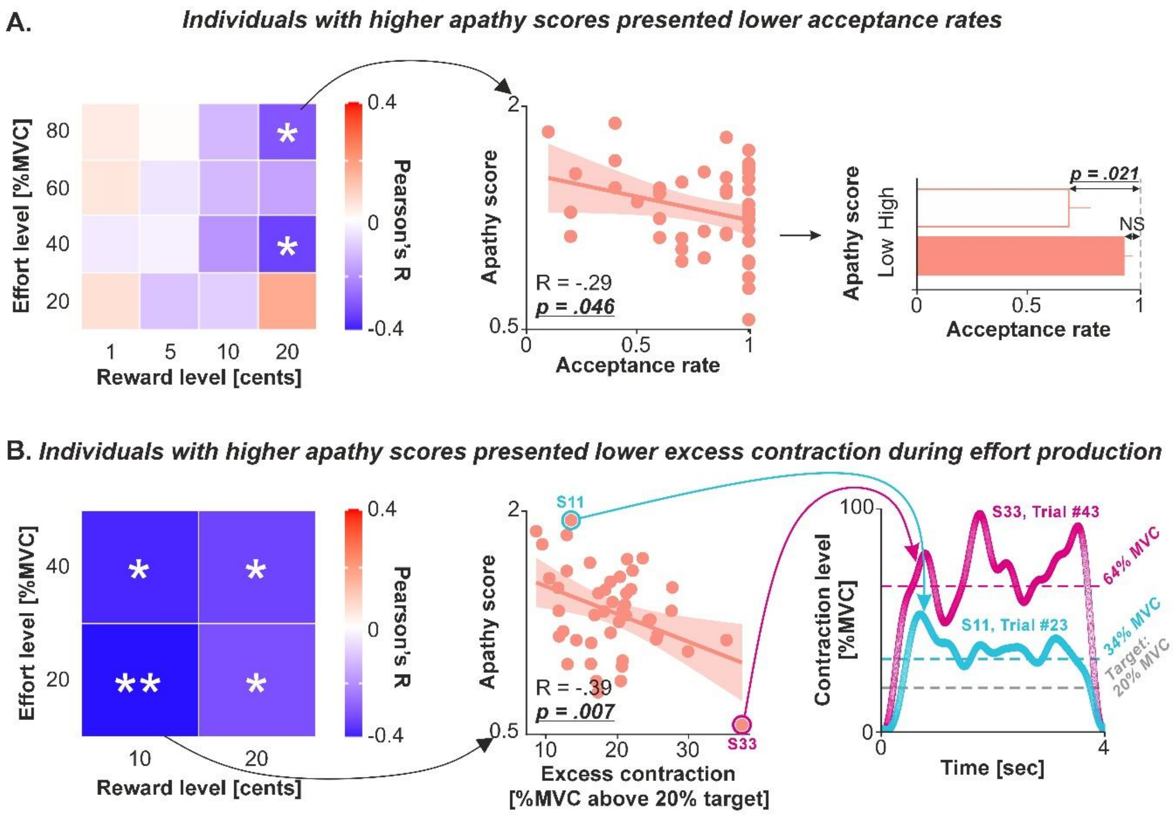

Supplementary Figure 2: Apathy scores correlated negatively with decision-making task metrics reflecting motivational drive during both decision and execution phases. A. Individuals with higher apathy scores presented a lower acceptance rate. The correlogram (right panel) displays Pearson correlation coefficients between apathy scores and acceptance rates across different combinations of effort and reward levels. Blue shades indicate negative correlations; stars indicate statistical significance ($p < .05$). A significant effect was observed for instance in trials with high effort (80% MVC) and high reward (20 cents), where a negative correlation was found ($R = -0.29$, $p = .046$; middle panel). This indicates that participants with higher apathy scores were less likely to accept these high-reward offers, suggesting reduced reward sensitivity. This relationship must be interpreted with caution due to ceiling effects, as several participants accepted nearly all high-reward trials (acceptance rate ~1). Nevertheless, the top left of the scatterplot illustrates that higher apathy scores were associated with lower acceptance in these trials. To further illustrate this effect, participants were split into low- and high-apathy groups (first and last quartiles, n = 10 per group). Low-apathy participants accepted 92% of these high-reward/high-effort offers (0.92 ± 0.04), while high-apathy participants accepted only 68% (0.68 ± 0.11). The acceptance rate of the high-apathy group was significantly lower than 1 ($t_9 = -2.78$, $p = .021$), and the between-group difference was marginally significant ($t_{18} = 1.96$, $p = .075$). These results suggest that higher apathy scores may be associated with a blunted behavioral drive to obtain high rewards in healthy subjects. **B. Individuals with higher apathy scores presented a lower excess contraction during effort production.** We analyzed a metric of excess contraction, defined as the amount of biceps contraction voluntarily produced beyond the required target (e.g., producing 35% MVC in a 20% MVC trial corresponds to 15% excess contraction), as an index of spontaneous effort investment. This analysis focused on a subset of trials with sufficient accepted responses across participants (20% and 40% MVC targets combined with 10 and 20 cent rewards). The correlogram (left panel) displays Pearson correlation coefficients between apathy scores and excess contraction across these conditions. Blue shading indicates negative correlations; asterisks denote statistical significance (*$p < .05$, **$p < .01$). Significant negative correlations were observed in all four effort-reward conditions (middle

panel shows correlation for 20% MVC / 10 cents: R = -0.39, p = .007;), indicating that participants with higher apathy levels produced smaller excess contractions. Single-trial examples from two representative participants with low vs. high apathy scores (S33 and S11, respectively) are also shown (left panel). Together with the acceptance rate findings, these results demonstrate that apathy is reflected not only in reduced willingness to engage in effortful actions but also in reduced voluntary effort investment during movement execution.

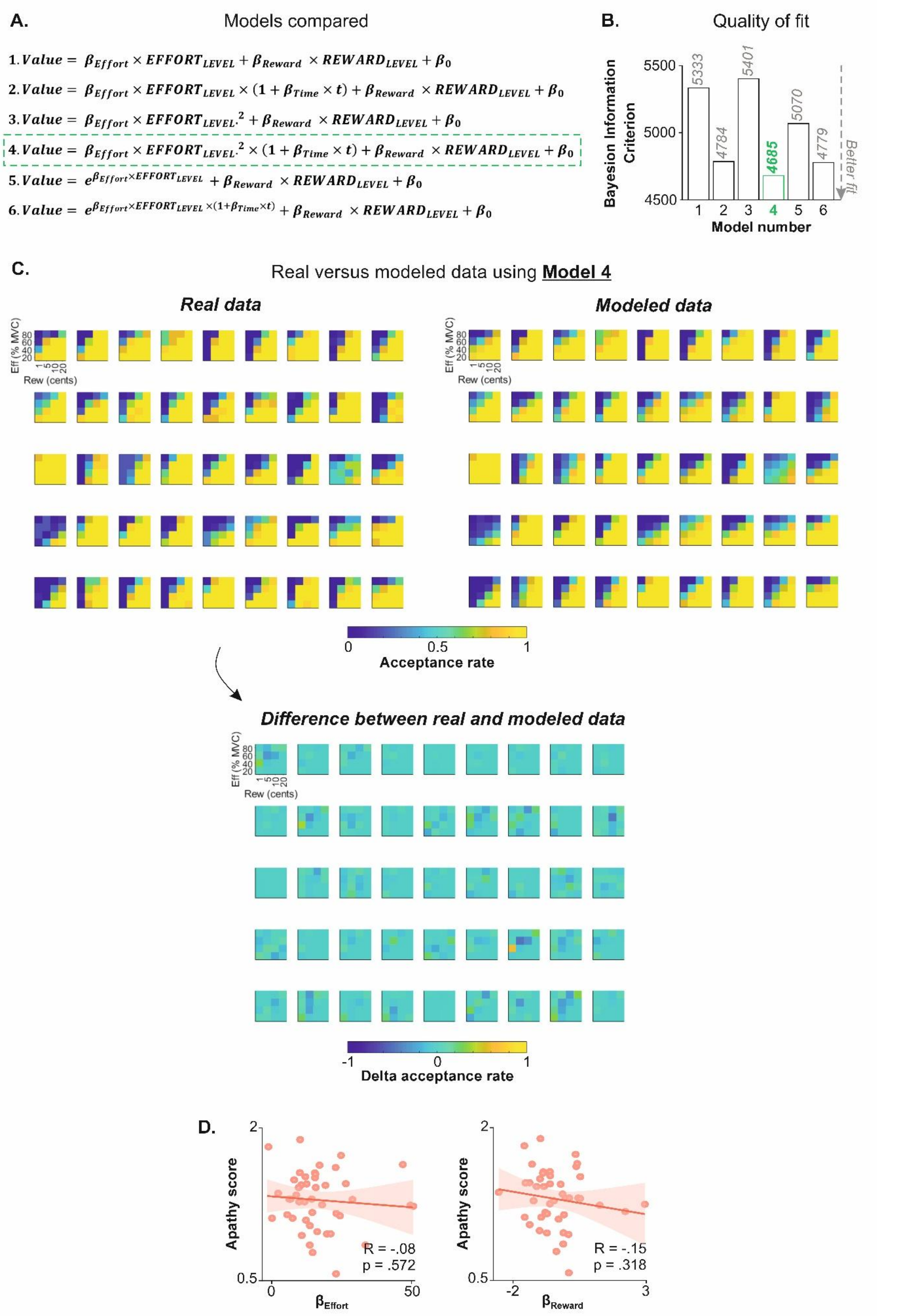

Supplementary Figure 3: Computational modeling of decision behavior. A. Model comparison. This panel shows the models tested in our study, following the same procedure

as LeHeron et al., 2018[2]. Based on prior research[2–4], we evaluated candidate models of value computation using standard methods – minimization of the Bayesian Information Criterion (BIC, see B) and visual inspection of model fits (see C and Figure 1.B). The models differed in cost functions and whether they included a β_{Time} parameter, which linearly modulated the cost term over trial number[4]. Specifically, Model 1 used a linear cost function without the β_{Time} parameter, Model 2 used a linear cost function with β_{Time}, Model 3 used a quadratic cost function without β_{Time}, Model 4 used a quadratic cost function with β_{Time}, Model 5 used an exponential cost function without β_{Time}, and Model 6 used an exponential cost function with β_{Time}. **B. Model fit quality.** This panel presents the BIC for each model. Model 4, featuring a quadratic cost function and a β_{Time} parameter, provided the best fit with the lowest BIC. This result aligns with previous studies[2,4], which also showed that quadratic cost functions with a β_{Time} parameter gave the best fit for this type of task. **C. Comparison of real and modeled data.** The top left maps show the actual acceptance rates (color-coded from low (blue) to high (yellow) acceptance rates) in the effort-based decision-making task for each of the 45 subjects. The x-axis represents reward levels (1–20 cents), and the y-axis represents effort levels (20–80% MVC). The top right maps show modeled data using Model 4 for each subject. The bottom maps display the differences between real and modeled data, demonstrating that Model 4 closely fits subject-level data. These results complement the average data fit shown in Figure 1.B. **D. Apathy scores did not significantly correlate with computational model parameters of effort and reward valuation.** Bayesian regression analyses indicated moderate evidence in favor of the null hypothesis, with Bayes Factors (BF_{01}) of 2.97 for β_{Effort} and 2.24 for β_{Reward}. These findings replicate previous observations and suggest that, while model-derived parameters provide a compact and interpretable summary of individual valuation differences, they may not fully capture inter-individual differences in subclinical apathy, particularly those that manifest under specific task conditions (*e.g.*, high reward trials, as shown in Supplementary Figure 2). This is likely because the modeling process, by design, emphasizes latent constructs across trial types, thereby averaging out trial-to-trial variability that might reflect subtle motivational differences linked to apathy. These results underscore the importance of combining model-based analyses, which capture general valuation tendencies, with model-free behavioral approaches, such as trial-by-trial acceptance rates, to more comprehensively assess motivational processes.

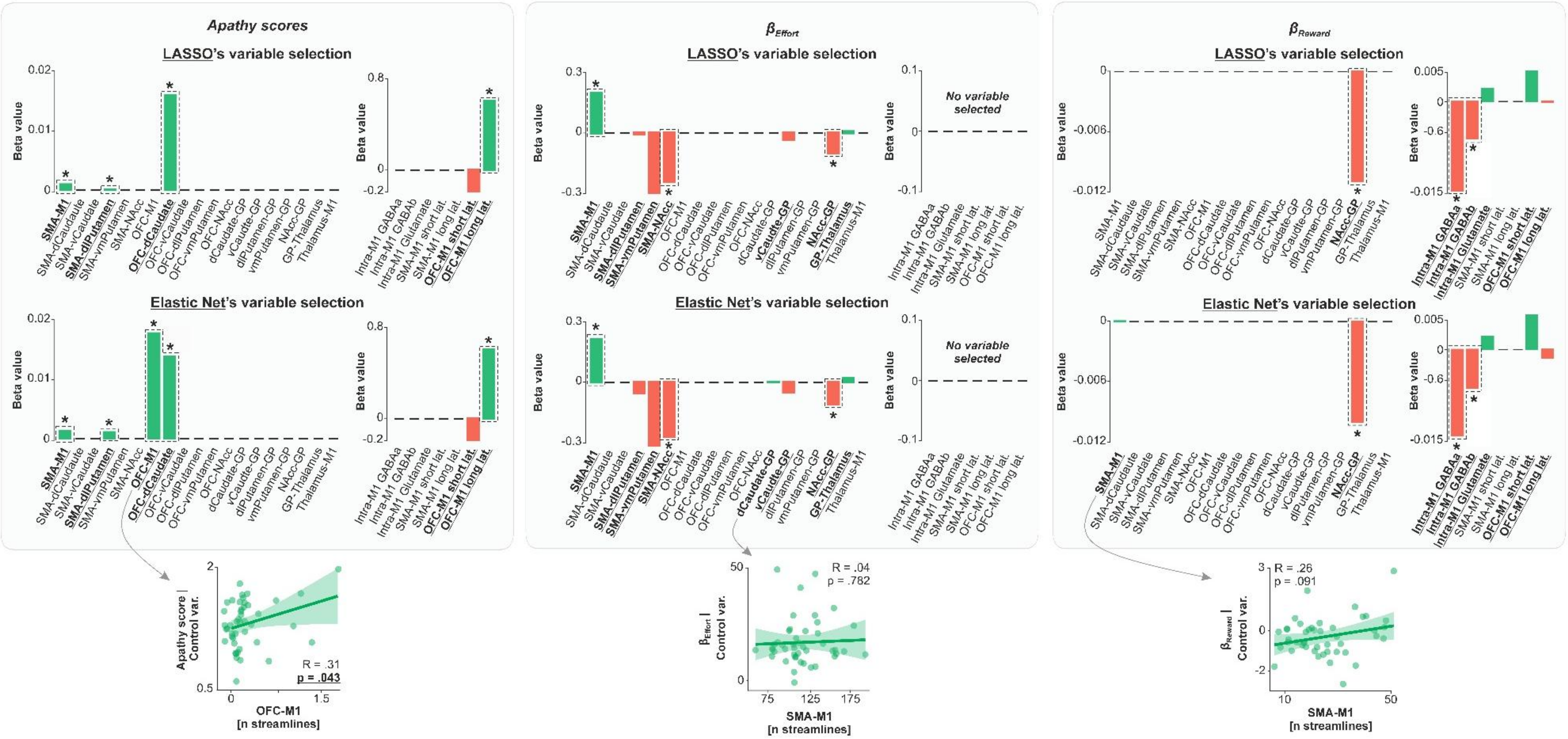

Supplementary Figure 4: An Elastic Net regression reproduced the same results as the LASSO regression. This figure presents the results of the Elastic Net regression analysis, which was conducted to address the potential risk of false negatives that may occur when using the conservative LASSO regression. As described in the main text, LASSO is a penalized least squares method that reduces the risk of false positives by selecting only relevant independent variables, excluding non-relevant variables by assigning them a regression coefficient of zero. While highly effective, LASSO's conservative nature may overlook relevant variables. To address this, we performed a less conservative Elastic Net regression. The results of the Elastic Net regression reproduced the main findings of the LASSO analysis, confirming the robustness of our results. Additionally, a complementary Bayes Factor Analysis provided further evidence for an absence of correlations between the apathy scores and the independent variables not selected by LASSO or Elastic Net regressions. Overall, across all analyses, LASSO regressions did not select 52 independent variables, providing beta coefficients equal to 0. The Elastic Net regression yielded highly similar results, excluding 49 independent variables. For β_{Effort} and β_{Reward}, the Elastic Net regression selected two additional variables (*i.e.*, the number of streamlines in dCaudate-GP and SMA-M1 tracts, respectively), but neither achieved statistical significance when assessed via partial correlation analysis (bottom panels of the figure; p = .782 and .091, respectively). For apathy scores, the Elastic Net regression identified the OFC-M1 tract as an additional independent variable covarying with apathy. Partial correlation analysis showed a statistically significant association between apathy scores and OFC-M1 structural connectivity (p = .043). However, this finding should be interpreted with caution: the correlation is weak, is based on limited variance due to the low anatomical density of projections from OFC to M1 (see x-axis values), and lacks support from effective connectivity data obtained using TMS, where no significant OFC-M1 correlation was found (see Supplementary Figure 6). Collectively, these results highlight the robustness of the primary findings, particularly regarding potential false negatives when using LASSO regression.

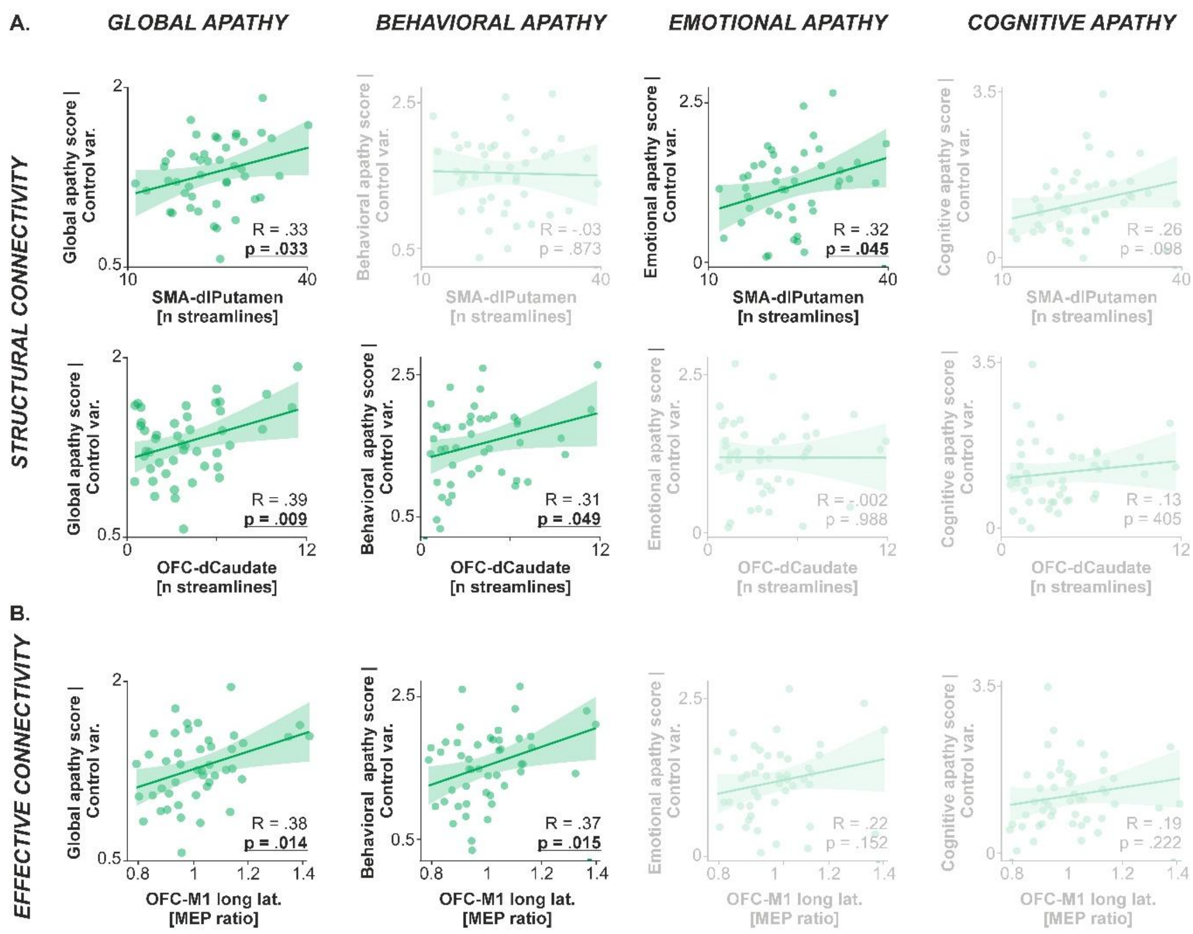

Supplementary Figure 5: Apathy subscores covary preferentially with connectivity in specific fronto-striatal and fronto-motor circuits. A. Structural connectivity. The leftmost column reproduces the main results presented in the manuscript, showing partial correlations between global apathy scores and streamline counts in two tracts identified by LASSO regression (OFC-dCaudate and SMA-dlPutamen). The three right columns present the same partial correlation analyses separately for behavioral, emotional, and cognitive apathy subscores, derived from the LARS-e questionnaire: Action Initiation subscale (behavioral apathy), Emotional Response subscale (emotional apathy), and Intellectual Curiosity – Novelty subscale (as a proxy for cognitive apathy). These analyses revealed that streamline count in the OFC-dCaudate tract was specifically associated with behavioral apathy (R = 0.31, p = .049), but not with emotional or cognitive apathy (R = –0.002, p = .998; R = 0.13, p = .405, respectively). In contrast, connectivity in the SMA-dlPutamen tract was positively associated with emotional apathy (R = 0.32, p = .045). SMA-M1 connectivity showed no significant association with any apathy subscore, suggesting that its contribution to global apathy may not reflect any single dimension in isolation. These findings support the notion that distinct fronto-striatal pathways are differentially involved in specific components of apathy. **B. Effective connectivity.** As observed for structural connectivity in A, partial correlation analysis for the long-latency OFC-M1 circuit showed a significant association with behavioral apathy (R = 0.37, p = .015), but not with emotional (R = 0.22, p = .152) or cognitive apathy (R = 0.19, p = .222), further implicating long-latency fronto-striato-motor pathways in deficits in action initiation. Note: Variables included in these analyses were selected via LASSO regression, reducing the likelihood of false positives; however, p-values reported here are uncorrected for multiple comparisons and should be interpreted with caution.

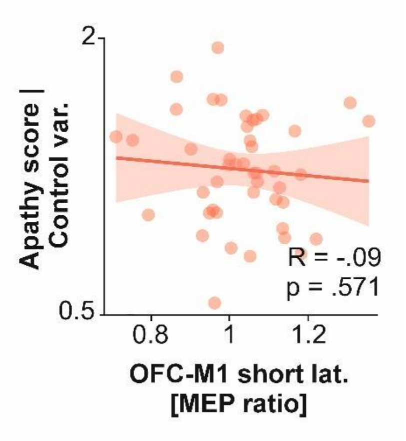

Supplementary Figure 6: Apathy scores are not significantly correlated with effective connectivity in the short-latency OFC-M1 circuit. The LASSO regression revealed two circuits (short- and long-latency OFC-M1) with non-zero coefficients (LASSO's β coefficients = -0.23 and 0.61, respectively; Figure 3). However, the partial correlation analysis showed that apathy scores were not significantly correlated with effective connectivity in the short-latency OFC-M1 circuit (R = -0.09, p = .571). The partial correlation only confirmed a significant positive correlation between apathy scores and the MEP ratio for the long-latency OFC-M1 circuit variable (R = 0.38, p = .014; Figure 3), indicating that higher apathy scores are associated with a stronger facilitatory influence of OFC on M1 specifically through this long-latency circuit.

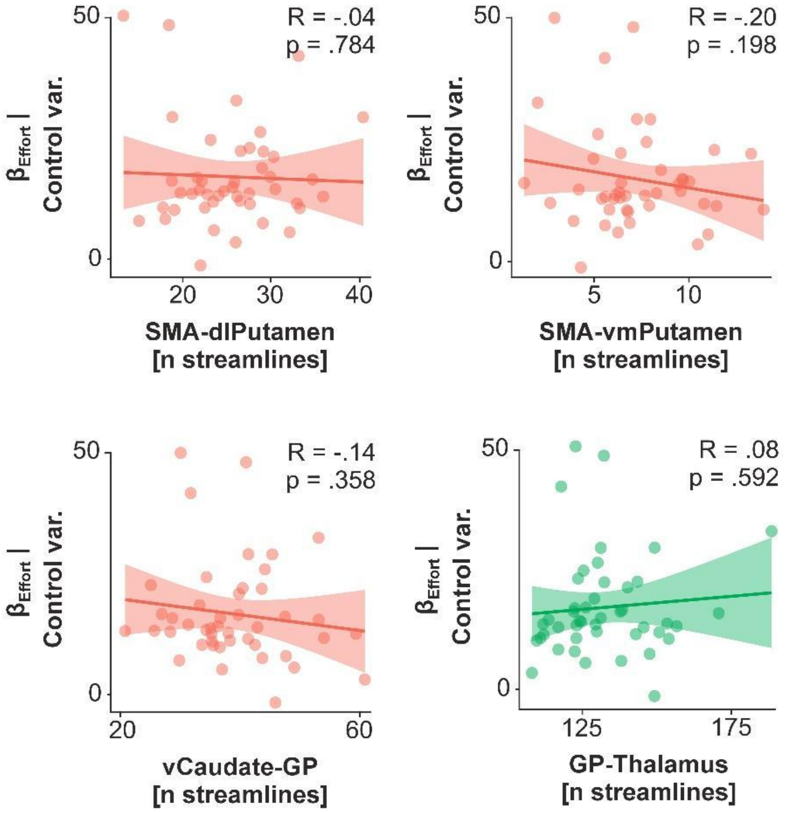

Supplementary Figure 7: Effort valuation is not significantly correlated with structural connectivity in SMA-dlPutamen, SMA-vmPutamen, vCaudate-GP and GP-Thalamus circuits. The LASSO regression identified seven tracts with non-zero coefficients: SMA-M1 (LASSO's $\beta = 0.21$), SMA-dlPutamen ($\beta = -0.02$), SMA-vmPutamen ($\beta = -0.31$), SMA-NAcc ($\beta = -0.25$), vCaudate-GP ($\beta = -0.04$), NAcc-GP ($\beta = -0.11$) and GP-Thalamus ($\beta = 0.02$; Figure 4). However, as evident in this figure, the partial correlation analysis showed that β_{Effort} was not significantly correlated with structural connectivity in SMA-dlPutamen, SMA-vmPutamen, vCaudate-GP and GP-Thalamus circuits (p-values range = [.198 .784]). Partial correlation analyses showed that correlations between β_{Effort} and the number of streamlines was only significant for three of these seven tracts, namely SMA-M1 ($R = 0.42$, $p = .006$), SMA-NAcc ($R = -0.32$, $p = .042$) and NAcc-GP circuits ($R = -0.41$, $p = .007$; Figure 4 in the main document).

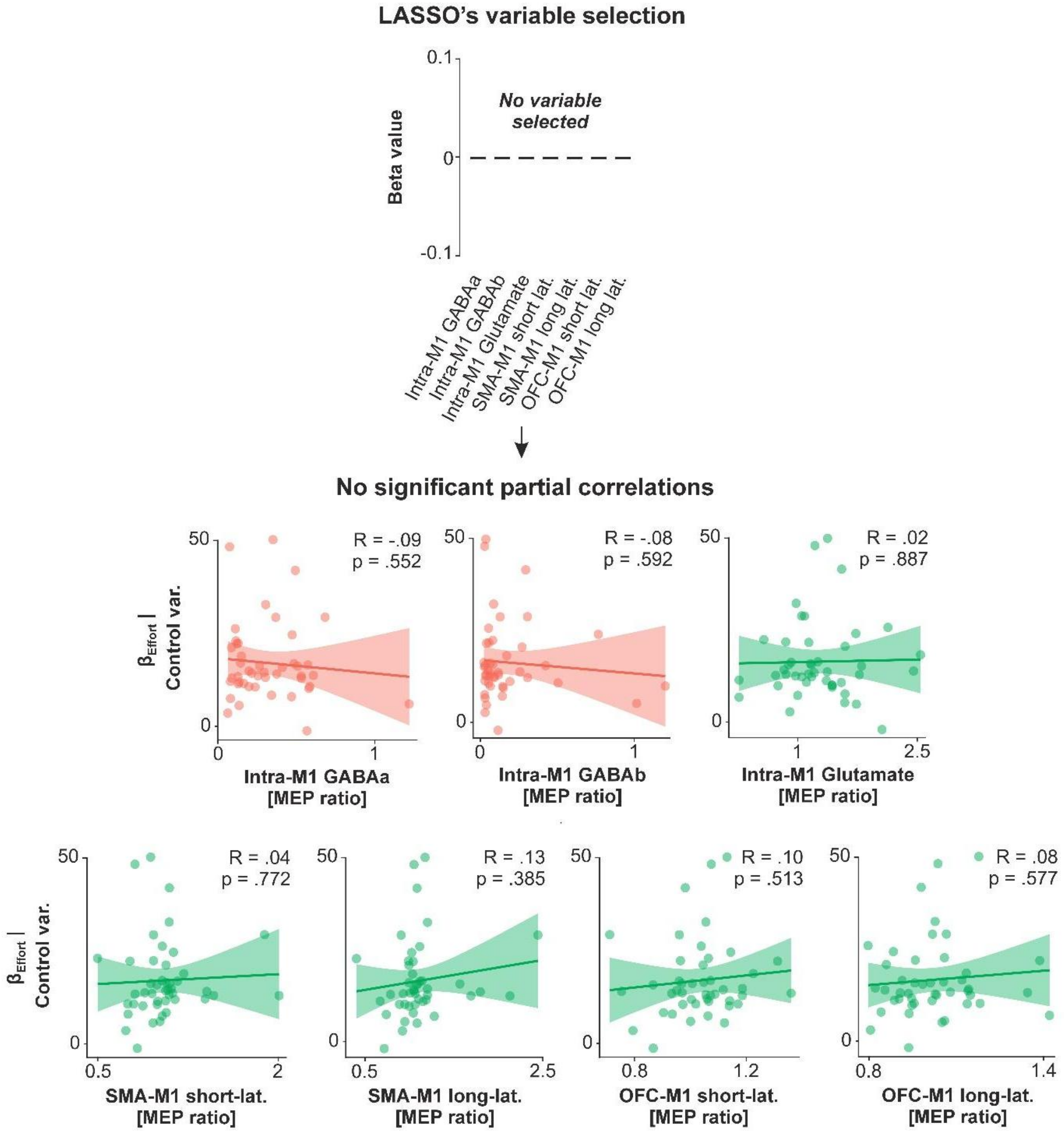

Supplementary Figure 8: Effort valuation was not significantly correlated with effective connectivity in the investigated circuits. We applied LASSO regression using β_{Effort} as the dependent variable and all TMS effective connectivity data (*i.e.*, all MEP ratios) as independent variables. The regression yielded zero coefficients for all circuits (*i.e.*, all $\beta = 0$), signifying a lack of association between β_{Effort} and effective connectivity in the investigated circuits (top graph). Partial correlations confirmed the absence of significant partial correlation between β_{Effort} and all effective connectivity data (p-values range = [.385 .887]).

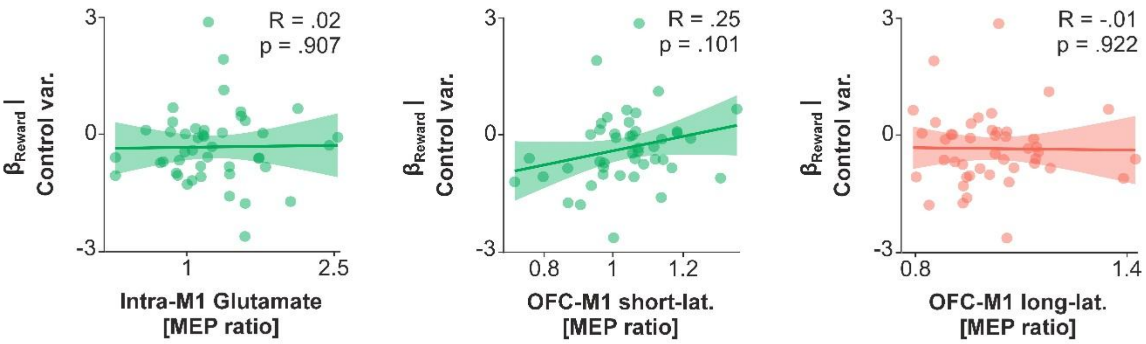

Supplementary Figure 9: Reward valuation is not significantly correlated with effective connectivity in intra-M1 glutamatergic, and OFC-M1 short- and long-latency circuits. The LASSO regression identified five circuits with non-zero coefficients (Figure 6): intra-M1 GABAa (LASSO's β = -0.015), intra-M1 GABAb (β = -0.006), intra-M1 glutamatergic (β = 0.002), short-latency OFC-M1 (β = 0.005) and long-latency OFC-M1 circuits (β = -0.0004). However, as evident in this figure, the partial correlation analysis showed that β_{Reward} was not significantly correlated with effective connectivity in intra-M1 glutamatergic, and OFC-M1 short- and long-latency circuits (p-values range = [.101 .922]). Partial correlation analyses showed that correlations between β_{Effort} and MEP ratios were only significant for two of these circuits, namely intra-M1 GABAa (R = -0.50, p = .0007) and intra-M1 GABAb circuits (R = -0.31, p = .047; Figure 6).

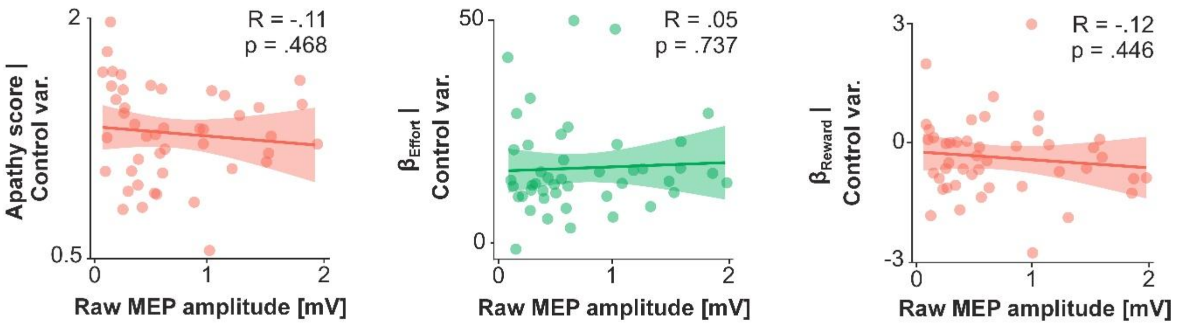

Supplementary Figure 10: Apathy scores, effort valuation and reward valuation are not linked to M1 net output. We performed partial correlations with apathy scores, β_{Effort}, and β_{Reward} as dependent variables and test MEP amplitudes (obtained with a single test stimulation over M1), a proxy for M1 net output[5,6], as the independent variable. The analysis showed no significant correlation between apathy scores (R = -0.11, p = .468), β_{Effort} (R = 0.05, p = .737), β_{Reward} (R = -0.12, p = .446) and test MEP amplitudes. Bayes Factor computation indicated a higher likelihood of no correlation (BF_{01} averaged 2.59 ± 0.34, range = [2.12 - 3.25]). Thus, despite correlations between apathy scores, effort valuation, reward valuation, and connectivity in circuits projecting to M1, these components of apathy are not linked to M1 net output alone. This analysis is also an important methodological control as it shows that the relationships between apathy scores, β_{Effort}, β_{Reward}, and MEP ratios are not due to associations with the test MEP amplitudes exploited to compute these ratios.

Supplementary information references

1. Bonnelle, V., Manohar, S., Behrens, T. & Husain, M. Individual Differences in Premotor Brain Systems Underlie Behavioral Apathy. *Cerebral Cortex* **26**, 807–819 (2016).
2. Le Heron, C. *et al.* Dysfunctional effort-based decision-making underlies apathy in genetic cerebral small vessel disease. *Brain* **141**, 3193–3210 (2018).
3. Prévost, C., Pessiglione, M., Météreau, E., Cléry-Melin, M. L. & Dreher, J. C. Separate valuation subsystems for delay and effort decision costs. *Journal of Neuroscience* **30**, 14080–14090 (2010).
4. Pessiglione, M., Vinckier, F., Bouret, S., Daunizeau, J. & Le Bouc, R. Why not try harder? Computational approach to motivation deficits in neuro-psychiatric diseases. *Brain* **141**, 629–650 (2018).
5. Derosiere, G., Vassiliadis, P. & Duque, J. Advanced TMS approaches to probe corticospinal excitability during action preparation. *NeuroImage* vol. 213 116746 Preprint at https://doi.org/10.1016/j.neuroimage.2020.116746 (2020).
6. Di Lazzaro, V., Rothwell, J. & Capogna, M. Noninvasive Stimulation of the Human Brain: Activation of Multiple Cortical Circuits. *Neuroscientist* vol. 24 246–260 Preprint at https://doi.org/10.1177/1073858417717660 (2018).
