# Supplementary material for: Fronto-motor circuits linked to effort-based decision-making and apathy in healthy subjects
Source: Commun Biol. 2025 Aug 30;8:1320. doi: 10.1038/s42003-025-08780-8 (PMC12398607; doi:10.1038/s42003-025-08780-8)
Supplement: Supplementary file 2 — Description of additional supplementary data [file 42003_2025_8780_MOESM2_ESM.docx]

**Description of additional supplementary data**

**Supplementary Data 1:** Average acceptance rates across the group of 45 subjects, used to generate the plot in Figure 1B.

**Supplementary Data 2:** Data used for the plots in Figure 2A and 2B, supporting the LASSO regression in 2A and the partial correlation analysis in 2B. Columns include gender, age, LARS (i.e., apathy) score, depression and anhedonia scores, and the number of streamlines for each tract of interest.

**Supplementary Data 3:** Data used for the plots in Figure 3A and 3B, supporting the LASSO regression in 3A and the partial correlation analysis in 3B. Columns include gender, age, LARS (i.e., apathy) score, depression and anhedonia scores, and MEP amplitudes for each circuit of interest.

**Supplementary Data 4:** Data used for the plots in Figure 4A and 4B, supporting the LASSO regression in 4A and the partial correlation analysis in 4B. Columns include gender, age, β_Effort_ values, depression and anhedonia scores, and the number of streamlines for each tract of interest.

**Supplementary Data 5:** Data used for the plots in Figure 5A and 5B, supporting the LASSO regression in 5A and the partial correlation analysis in 5B. Columns include gender, age, β_Reward_ values, depression and anhedonia scores, and the number of streamlines for each tract of interest.

**Supplementary Data 6:** Data used for the plots in Figure 6A and 6B, supporting the LASSO regression in 5A and the partial correlation analysis in 6B. Columns include gender, age, β_Reward_ values, depression and anhedonia scores, and MEP amplitudes for each circuit of interest.
