# Supplementary material for: Fronto-motor circuits linked to effort-based decision-making and apathy in healthy subjects
Source: Commun Biol. 2025 Aug 30;8:1320. doi: 10.1038/s42003-025-08780-8 (PMC12398607; doi:10.1038/s42003-025-08780-8)
Supplement: Supplementary file 9 — Reporting summary [file 42003_2025_8780_MOESM9_ESM.pdf]

Corresponding author(s): Dr Gerard Derosiere

Last updated by author(s): Jul 15, 2025

Reporting Summary

Nature Portfolio wishes to improve the reproducibility of the work that we publish. This form provides structure for consistency and transparency in reporting. For further information on Nature Portfolio policies, see our Editorial Policies and the Editorial Policy Checklist.

Statistics

For all statistical analyses, confirm that the following items are present in the figure legend, table legend, main text, or Methods section.

n/a	Confirmed	
☐	☒	The exact sample size (*n*) for each experimental group/condition, given as a discrete number and unit of measurement
☐	☒	A statement on whether measurements were taken from distinct samples or whether the same sample was measured repeatedly
☐	☒	The statistical test(s) used AND whether they are one- or two-sided<br>*Only common tests should be described solely by name; describe more complex techniques in the Methods section.*
☐	☒	A description of all covariates tested
☐	☒	A description of any assumptions or corrections, such as tests of normality and adjustment for multiple comparisons
☐	☒	A full description of the statistical parameters including central tendency (e.g. means) or other basic estimates (e.g. regression coefficient) AND variation (e.g. standard deviation) or associated estimates of uncertainty (e.g. confidence intervals)
☐	☒	For null hypothesis testing, the test statistic (e.g. *F*, *t*, *r*) with confidence intervals, effect sizes, degrees of freedom and *P* value noted<br>*Give P values as exact values whenever suitable.*
☐	☒	For Bayesian analysis, information on the choice of priors and Markov chain Monte Carlo settings
☒	☐	For hierarchical and complex designs, identification of the appropriate level for tests and full reporting of outcomes
☐	☒	Estimates of effect sizes (e.g. Cohen's *d*, Pearson's *r*), indicating how they were calculated

Our web collection on statistics for biologists contains articles on many of the points above.

Software and code

Policy information about availability of computer code

Data collection: We used custom-made (Matlab 2018a) scripts to collect the effort-based decision-making data.

Data analysis: Computational modelling of decision behavior: Matlab 2018a.
Calculation of neuropsychological scores: R version 4.3.1.
Streamline tractography: Python XX.
Statistical analyses including LASSO regression, partial correlations and final plots: R version 4.3.1.

For manuscripts utilizing custom algorithms or software that are central to the research but not yet described in published literature, software must be made available to editors and reviewers. We strongly encourage code deposition in a community repository (e.g. GitHub). See the Nature Portfolio guidelines for submitting code & software for further information.

Data

Policy information about availability of data

All manuscripts must include a data availability statement. This statement should provide the following information, where applicable:

- Accession codes, unique identifiers, or web links for publicly available datasets
- A description of any restrictions on data availability
- For clinical datasets or third party data, please ensure that the statement adheres to our policy

Data availability:

All data necessary to generate the main results and figures are available in the OSF repository (https://osf.io/a59mt/). The Brainnetome atlas was used for defining our regions of interest in the tractography analysis and can be downloaded from: http://atlas.brainnetome.org/.

Research involving human participants, their data, or biological material

Policy information about studies with human participants or human data. See also policy information about sex, gender (identity/presentation), and sexual orientation and race, ethnicity and racism.

Reporting on sex and gender	We recruited a total of 31 women and 14 men. Sex was determined based on self-reporting. We used the factor sex as a control variables in our partial analyses.
Reporting on race, ethnicity, or other socially relevant groupings	We did not determine race, ethnicity, or any other socially relevant grouping variables.
Population characteristics	The study initially involved 50 healthy human subjects. However, the experimental design consisted of two sessions and 3 out of the 50 subjects did not attend the second session. Additionally, tractography analysis could not be performed on 2 subjects due to corrupted MRI data. Consequently, the results presented in this article focus on the data of the 45 remaining subjects (25.1 ± 0.8 years old, 31 females, 14 males).<br>All subjects were right-handed according to the Edinburgh Questionnaire (Oldfield, 1971). None of the participants had any neurological disorder or history of psychiatric illness or drug or alcohol abuse, or were on any drug treatments that could influence performance.
Recruitment	Participants were recruited from the Research Participant Pool at the Institute of Neuroscience of UCLouvain.<br>Selection bias: Young subjects were recruited to a significant part within the university community through verbal or written advertisements. This entails that a disproportionately high number of subjects with a high level of education were recruited. To minimize the impact of this selection bias, we distributed the advertisement also at other public places.
Ethics oversight	The protocol was approved by the institutional review board of the Université catholique de Louvain, Brussels, Belgium, and required written informed consent.

Note that full information on the approval of the study protocol must also be provided in the manuscript.

Field-specific reporting

Please select the one below that is the best fit for your research. If you are not sure, read the appropriate sections before making your selection.

☒ Life sciences ☐ Behavioural & social sciences ☐ Ecological, evolutionary & environmental sciences

For a reference copy of the document with all sections, see nature.com/documents/nr-reporting-summary-flat.pdf

Life sciences study design

All studies must disclose on these points even when the disclosure is negative.

Sample size	The sample size for this study was carefully determined to align with prior research demonstrating significant associations between brain structure or function and subclinical apathy (e.g., Bonnelle et al., 2016, Cerebral Cortex, n = 37). Using the average correlation coefficient (R = 0.42) reported by Bonnelle et al. (2016) for connectivity and apathy scores in healthy individuals, we calculated that a minimum of 42 participants would be required to reliably detect similar effects with an alpha level of 0.05 and a statistical power of 0.8. To account for potential dropouts, we initially targeted a sample size of 50. Our final sample of 45 thus provides a margin to ensure adequate power.
Data exclusions	The study initially involved 50 healthy human subjects recruited from the Research Participant Pool at the Institute of Neuroscience of UCLouvain. However, the experimental design consisted of two sessions and 3 out of the 50 subjects did not attend the second session. Additionally, tractography analysis could not be performed on 2 subjects due to corrupted MRI data. Consequently, the results presented in this article focus on the data of the 45 remaining subjects (25.1 ± 0.8 years old, 31 females, 14 males).<br><br>MEP data: Trials in which the root mean square of the EMG signal exceeded 200 µV before stimulation (i.e., -250 to -50 ms from the pulse) were excluded from the analysis. Second, MEPs with an amplitude exceeding 3 SD around the mean within a given condition were also discarded to reduce MEP variability effects. After applying this cleaning procedure, we retained an average of 22 trials for each condition. The minimum and maximum numbers of MEPs per condition was 17 and 24, respectively.
Replication	The absence of association between the independent and dependent variables identified by the LASSO regression was further confirmed through Bayesian analysis (see page 8). Additionally, the exact same variable selection as the one operated by the LASSO regression was obtained using a less conservative Elastic Net regression, replicating exactly the same results using a different statistical approach and confirming the absence of false negatives (Supplementary Figure 4). Finally, all variables selected by the LASSO regression were also chosen using a stepwise regression approach, again confirming that the findings are independent of the analytical method used.
Randomization	Participant randomization: The study did not involve any group comparison and thus did not required any participant randomization.<br>EBDM task: Participants performed 5 blocks of 32 trials, each block including two repetitions of each of the 16 effort/reward conditions. There were therefore 160 trials in total, with 10 trials per condition. Conditions were presented in a randomized manner within each block.<br>TMS: The pTMS protocol comprised trials with single-pulse (i.e., TS only) and paired-pulse TMS (i.e., CS+TS with the intervals mentioned

above), occurring in a randomized order.

Blinding: Experimenters were not aware of the apathy scores as well as effort and reward valuation of the participants during the acquisition of the MRI and TMS data, ensuring that they could not influence the relationships between apathy scores as well as effort and reward valuation and MRI and TMS data reported in the manuscript.

Reporting for specific materials, systems and methods

We require information from authors about some types of materials, experimental systems and methods used in many studies. Here, indicate whether each material, system or method listed is relevant to your study. If you are not sure if a list item applies to your research, read the appropriate section before selecting a response.

Materials & experimental systems

n/a	Involved in the study
☒	☐ Antibodies
☒	☐ Eukaryotic cell lines
☒	☐ Palaeontology and archaeology
☒	☐ Animals and other organisms
☒	☐ Clinical data
☒	☐ Dual use research of concern
☒	☐ Plants

Methods

n/a	Involved in the study
☒	☐ ChIP-seq
☒	☐ Flow cytometry
☐	☒ MRI-based neuroimaging

Plants

Seed stocks: NA

Novel plant genotypes: NA

Authentication: NA

Magnetic resonance imaging

Experimental design

Design type: Resting-state.

Design specifications: One resting-state session per subject.

Behavioral performance measures: NA.

Acquisition

Imaging type(s): T1, resting-state functional MRI, diffusion-weigthed imaging (DWI).

Field strength: 3T

Sequence & imaging parameters: A 3D T1-weighted image was acquired for each participant on a 3T GE SIGNATM Premier scanner (GE Healthcare, Chicago, IL) with the following parameters: Echo Time (TE) = 2.96 ms, Repetition Time (TR) = 2238.93 ms, Inversion Time (TI) = 900 ms, 170 slices, slice thickness: 1 mm,in-plane Field Of View (FOV): 256 × 256 mm², matrix size: 256x256, 1 mm isotropic.

Area of acquisition: Whole brain.

Diffusion MRI: ☒ Used ☐ Not used

Parameters: All participants also underwent a diffusion-weighted MRI scan with the following parameters: TR = 7289 ms, TE = 57.1 ms, 70 slices, slice thickness: 2 mm,, in-plane FOV: 220 × 220 mm², matrix size: 110x110, 2 mm isotropic voxels, 64 gradients at b = 1000 s/mm² , and one reference b0 image.

Preprocessing

Preprocessing software

Preprocessing of the diffusion data was performed using the Elikopy pipeline. This included brain extraction using FreeSurfer (mri_synth_strip), thermal denoising using MRtrix3 (dwidenoise), and correction for susceptibility-induced distortions, eddy-current distortions, and head-motion using FSL (v6.0.7.8). Due to the unavailability of an unweighted diffusion image with reversed phase-encoding, Synb0-DISCO was used to synthesize a distortion-free unweighted diffusion image from the subject's T1-weighted image, which served as input for the distortion correction steps within FSL.

Normalization

Linear registration of metrics from diffusion MRI space (using the brain-masked b=0 image) to the subject's T1-weighted structural space (using the brain-masked T1w image) was performed using ANTs (Advanced Normalization Tools). Subsequently, a non-linear registration was performed using ANTs to warp the subject's brain-masked T1w image to the MNI152 standard space.

Normalization template

Data were normalized to the ICBM 152 non-linear 2009c asymmetric brain template.

Noise and artifact removal

Artifacts and structured noise were addressed during the preprocessing stage using multiple tools:
1. Thermal noise: Mitigated using MRtrix3's denoising algorithms.
2. Susceptibility-induced distortions: Corrected using FSL tools, informed by a synthetic distortion-free b=0 image generated via Synb0-DISCO from the T1w image.
3. Eddy-current distortions and subject head motion: Simultaneously corrected using FSL's eddy tool. This process models and corrects artifacts arising from gradient switching and participant movement. Motion parameters estimated by eddy were used within this correction framework.
No additional regressors for physiological noise (e.g., cardiac, respiratory signals) or specific nuisance tissue signals were explicitly modeled beyond the corrections performed by the aforementioned tools.

Volume censoring

No explicit volume censoring was performed after the preprocessing pipeline. Quality control and artifact mitigation were primarily handled by the ElikoPy pipeline's integrated tools, including FSL's eddy. The eddy tool incorporates functionality to detect and replace slices corrupted by excessive inter-slice motion or signal dropout based on expected diffusion signal properties, rather than removing entire volumes.

Statistical modeling & inference

Model type and settings

We selected pairs of structures from the streamline tractography matrices that comprised the fronto-BG-M1 and fronto-M1 circuits of interest in the present study. Specifically, we examined the following structures: the SMA, the OFC, the M1, and all structures of the BG that were identifiable using the Brainnetome atlas. These included the dorsal Caudate (dCaudate), the ventral Caudate (vCaudate), the dorsolateral Putamen (dlPutamen), the ventromedial Putamen (vmPutamen), and the Nucleus Accumbens (NAcc). To ensure the completeness of the circuits, we also incorporated the motor part of the thalamus. We extracted the number of streamlines for the following 19 pairs of structures: SMA-M1, SMA-dCaudate, SMA-vCaudate, SMA-dlPutamen, SMA-vmPutamen, SMA-NAcc, OFC-M1, OFC-dCaudate, OFC-vCaudate, OFC-dlPutamen, OFC-vmPutamen, OFC-NAcc, dCaudate-GP, vCaudate-GP, dlPutamen-GP, vmPutamen-GP, NAcc-GP, GP-Thalamus, and Thalamus-M1.

We then applied a LASSO regression model and partial regressions on these independent vairables to test our hypotheses (see "Effects tested" below).

Effect(s) tested

Our study aimed to identify which independent variables quantifying structural connectivity (number of streamlines in 19 tracts) and effective connectivity (MEP ratios) are associated with three dependent variables: apathy scores, βEffort and βReward. We employed a conservative two-step statistical approach. First, we used Least Absolute Shrinkage and Selection Operator (LASSO) regression, a penalized least squares method that selects relevant independent variables. LASSO can yield some regression coefficients (β coefficients) as zero. Only independent variables with non-zero regression coefficients are considered as associated with the dependent variable and thus selected. Second, we performed partial correlations between the dependent variables and the independent variables selected by the LASSO regression. This was done to control for multiple potentially confounding variables that may covary with apathy and effort-based decision-making processes (i.e., age, gender, depression, and anhedonia), ensuring that the associations we identified between apathy scores, βEffort, and βReward, on the one hand, and structural or effective connectivity, on the other, were truly specific to the dependent variable considered.

Specify type of analysis: ☐ Whole brain ☒ ROI-based ☐ Both

Anatomical location(s)

19 pairs of structures slected based on a priori hypotheses: SMA-M1, SMA-dCaudate, SMA-vCaudate, SMA-dlPutamen, SMA-vmPutamen, SMA-NAcc, OFC-M1, OFC-dCaudate, OFC-vCaudate, OFC-dlPutamen, OFC-vmPutamen, OFC-NAcc, dCaudate-GP, vCaudate-GP, dlPutamen-GP, vmPutamen-GP, NAcc-GP, GP-Thalamus, and Thalamus-M1.

Statistic type for inference

(See Eklund et al. 2016)

NA.

Correction

NA.

Models & analysis

n/a	Involved in the study
☒	☐ Functional and/or effective connectivity
☒	☐ Graph analysis
☒	☐ Multivariate modeling or predictive analysis
